# Supplementary figures and images for: The modeling and condition analysis of nondestructive testing based on ESPI for internal defects of materials (part 2 of 2)
Source: PLoS One. 2025 Jul 1;20(7):e0327318. doi: 10.1371/journal.pone.0327318 (PMC12212563; doi:10.1371/journal.pone.0327318)

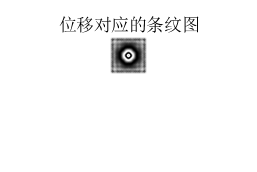

Supplement: S1 Dataset — (ZIP) [file pone.0327318.s001.zip › Minimal data set/Data for Table 6-Table 10_Thermal loading/2500-3S-50S/D15-S05.bmp]

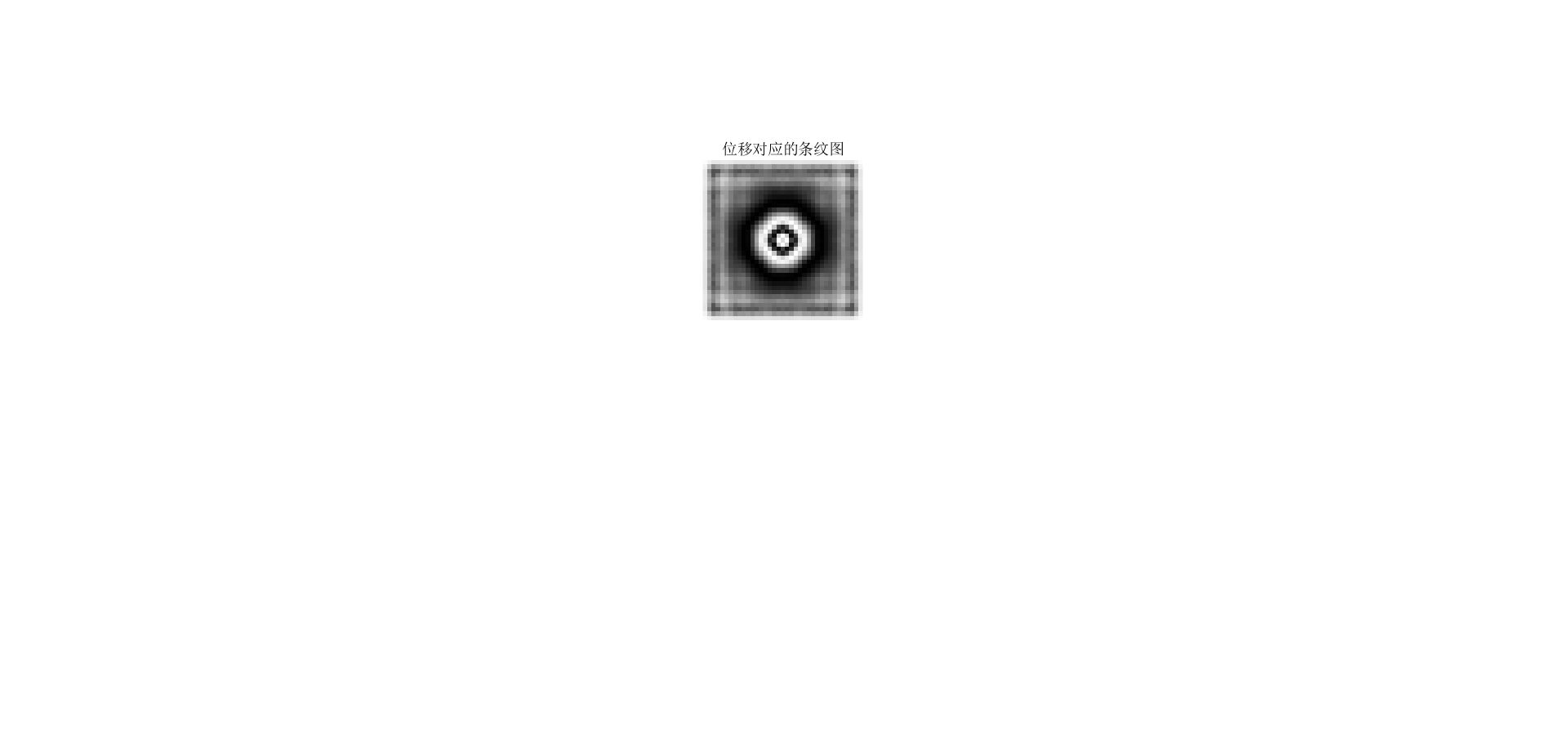

Supplement: S1 Dataset — (ZIP) [file pone.0327318.s001.zip › Minimal data set/Data for Table 6-Table 10_Thermal loading/2500-3S-50S/D15S05.bmp]

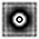

Supplement: S1 Dataset — (ZIP) [file pone.0327318.s001.zip › Minimal data set/Data for Table 6-Table 10_Thermal loading/2500-3S-50S/D15S05.png]

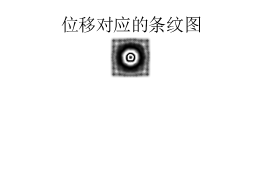

Supplement: S1 Dataset — (ZIP) [file pone.0327318.s001.zip › Minimal data set/Data for Table 6-Table 10_Thermal loading/2500-3S-50S/D16-S05.bmp]

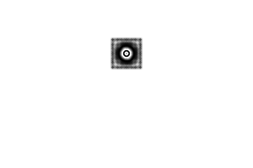

Supplement: S1 Dataset — (ZIP) [file pone.0327318.s001.zip › Minimal data set/Data for Table 6-Table 10_Thermal loading/2500-3S-50S/D16S05.bmp]

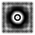

Supplement: S1 Dataset — (ZIP) [file pone.0327318.s001.zip › Minimal data set/Data for Table 6-Table 10_Thermal loading/2500-3S-50S/D16S05.png]

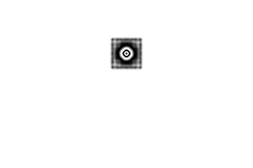

Supplement: S1 Dataset — (ZIP) [file pone.0327318.s001.zip › Minimal data set/Data for Table 6-Table 10_Thermal loading/2500-3S-50S/D17S05.bmp]

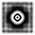

Supplement: S1 Dataset — (ZIP) [file pone.0327318.s001.zip › Minimal data set/Data for Table 6-Table 10_Thermal loading/2500-3S-50S/D17S05.png]

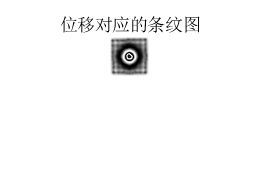

Supplement: S1 Dataset — (ZIP) [file pone.0327318.s001.zip › Minimal data set/Data for Table 6-Table 10_Thermal loading/2500-3S-50S/D18-S05.bmp]

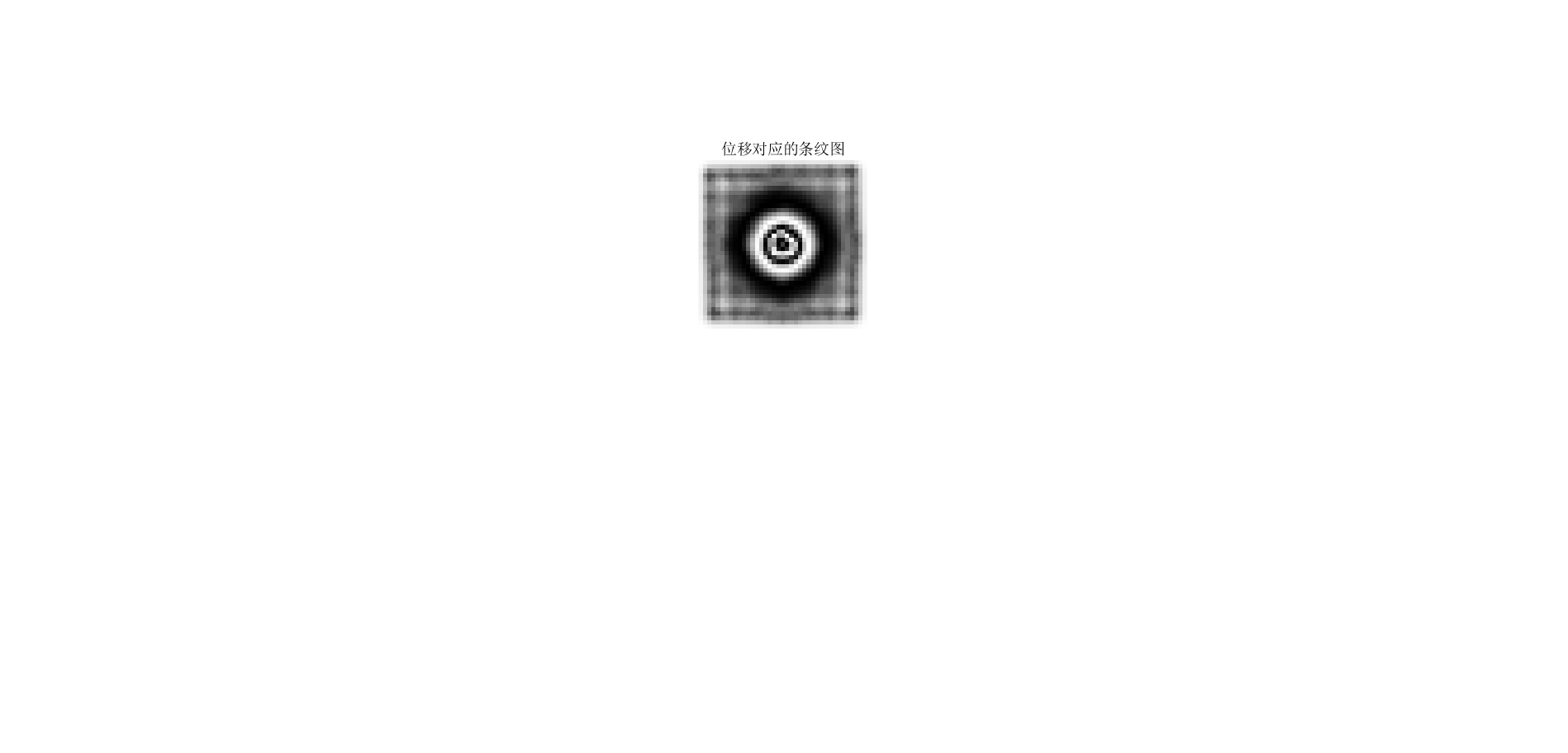

Supplement: S1 Dataset — (ZIP) [file pone.0327318.s001.zip › Minimal data set/Data for Table 6-Table 10_Thermal loading/2500-3S-50S/D18S05.bmp]

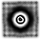

Supplement: S1 Dataset — (ZIP) [file pone.0327318.s001.zip › Minimal data set/Data for Table 6-Table 10_Thermal loading/2500-3S-50S/D18S05.png]

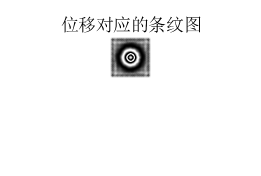

Supplement: S1 Dataset — (ZIP) [file pone.0327318.s001.zip › Minimal data set/Data for Table 6-Table 10_Thermal loading/2500-3S-50S/D19S05.bmp]

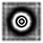

Supplement: S1 Dataset — (ZIP) [file pone.0327318.s001.zip › Minimal data set/Data for Table 6-Table 10_Thermal loading/2500-3S-50S/D19S05.png]

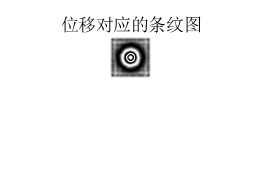

Supplement: S1 Dataset — (ZIP) [file pone.0327318.s001.zip › Minimal data set/Data for Table 6-Table 10_Thermal loading/2500-3S-50S/D20-S05.bmp]

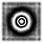

Supplement: S1 Dataset — (ZIP) [file pone.0327318.s001.zip › Minimal data set/Data for Table 6-Table 10_Thermal loading/2500-3S-50S/D20S05.png]

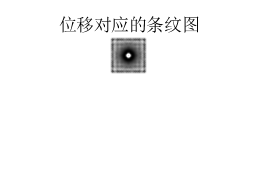

Supplement: S1 Dataset — (ZIP) [file pone.0327318.s001.zip › Minimal data set/Data for Table 6-Table 10_Thermal loading/2500-3S-50S/D9S05.jpg]

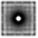

Supplement: S1 Dataset — (ZIP) [file pone.0327318.s001.zip › Minimal data set/Data for Table 6-Table 10_Thermal loading/2500-3S-50S/D9S05.png]

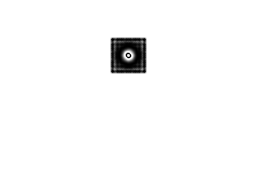

Supplement: S1 Dataset — (ZIP) [file pone.0327318.s001.zip › Minimal data set/Data for Table 6-Table 10_Thermal loading/3750-3-50/D10S05.bmp]

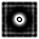

Supplement: S1 Dataset — (ZIP) [file pone.0327318.s001.zip › Minimal data set/Data for Table 6-Table 10_Thermal loading/3750-3-50/D10S05.png]

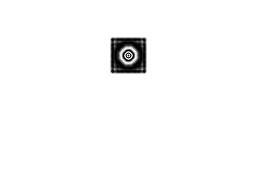

Supplement: S1 Dataset — (ZIP) [file pone.0327318.s001.zip › Minimal data set/Data for Table 6-Table 10_Thermal loading/3750-3-50/D15S05.bmp]

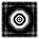

Supplement: S1 Dataset — (ZIP) [file pone.0327318.s001.zip › Minimal data set/Data for Table 6-Table 10_Thermal loading/3750-3-50/D15S05.png]

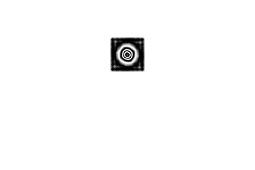

Supplement: S1 Dataset — (ZIP) [file pone.0327318.s001.zip › Minimal data set/Data for Table 6-Table 10_Thermal loading/3750-3-50/D20S05(1.57).bmp]

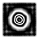

Supplement: S1 Dataset — (ZIP) [file pone.0327318.s001.zip › Minimal data set/Data for Table 6-Table 10_Thermal loading/3750-3-50/D20S05.png]

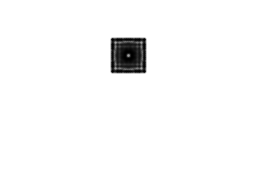

Supplement: S1 Dataset — (ZIP) [file pone.0327318.s001.zip › Minimal data set/Data for Table 6-Table 10_Thermal loading/3750-3-50/D5S05.bmp]

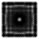

Supplement: S1 Dataset — (ZIP) [file pone.0327318.s001.zip › Minimal data set/Data for Table 6-Table 10_Thermal loading/3750-3-50/D5S05.png]

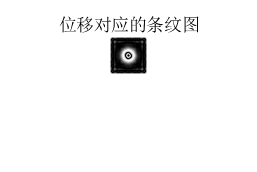

Supplement: S1 Dataset — (ZIP) [file pone.0327318.s001.zip › Minimal data set/Data for Table 6-Table 10_Thermal loading/5000-3S-50S/5000D10S05.bmp]

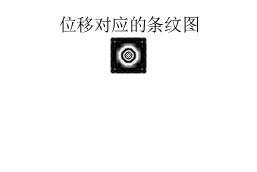

Supplement: S1 Dataset — (ZIP) [file pone.0327318.s001.zip › Minimal data set/Data for Table 6-Table 10_Thermal loading/5000-3S-50S/5000D15S05.bmp]
