# Supplementary figures and images for: The modeling and condition analysis of nondestructive testing based on ESPI for internal defects of materials (part 1 of 2)
Source: PLoS One. 2025 Jul 1;20(7):e0327318. doi: 10.1371/journal.pone.0327318 (PMC12212563; doi:10.1371/journal.pone.0327318)

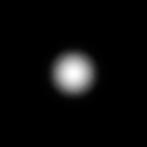

Supplement: S1 Dataset — (ZIP) [file pone.0327318.s001.zip › Minimal data set/Data for Figure 2-6/displacement_after interpolation.jpg]

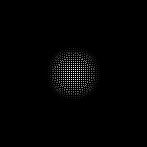

Supplement: S1 Dataset — (ZIP) [file pone.0327318.s001.zip › Minimal data set/Data for Figure 2-6/displacement_before interpolation.jpg]

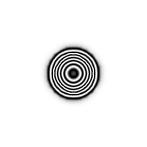

Supplement: S1 Dataset — (ZIP) [file pone.0327318.s001.zip › Minimal data set/Data for Figure 2-6/fringe_after interpolation.jpg]

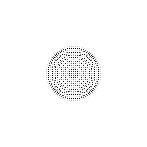

Supplement: S1 Dataset — (ZIP) [file pone.0327318.s001.zip › Minimal data set/Data for Figure 2-6/fringe_before interpolation.jpg]

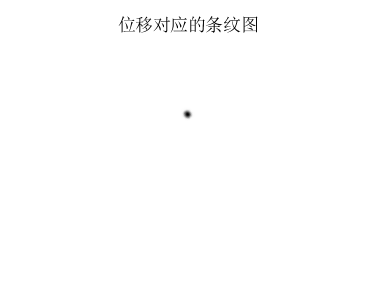

Supplement: S1 Dataset — (ZIP) [file pone.0327318.s001.zip › Minimal data set/Data for Table 2-Table 5_Vacuum Loading/0080046/0080046条纹图/008004610000.bmp]

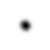

Supplement: S1 Dataset — (ZIP) [file pone.0327318.s001.zip › Minimal data set/Data for Table 2-Table 5_Vacuum Loading/0080046/0080046条纹图/008004610000.png]

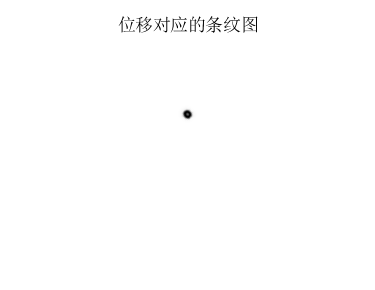

Supplement: S1 Dataset — (ZIP) [file pone.0327318.s001.zip › Minimal data set/Data for Table 2-Table 5_Vacuum Loading/0080046/0080046条纹图/008004620000.bmp]

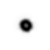

Supplement: S1 Dataset — (ZIP) [file pone.0327318.s001.zip › Minimal data set/Data for Table 2-Table 5_Vacuum Loading/0080046/0080046条纹图/008004620000.png]

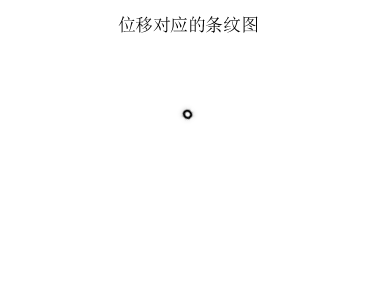

Supplement: S1 Dataset — (ZIP) [file pone.0327318.s001.zip › Minimal data set/Data for Table 2-Table 5_Vacuum Loading/0080046/0080046条纹图/008004630000.bmp]

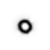

Supplement: S1 Dataset — (ZIP) [file pone.0327318.s001.zip › Minimal data set/Data for Table 2-Table 5_Vacuum Loading/0080046/0080046条纹图/008004630000.png]

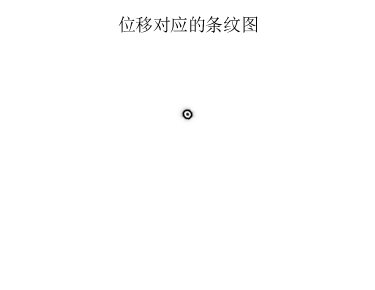

Supplement: S1 Dataset — (ZIP) [file pone.0327318.s001.zip › Minimal data set/Data for Table 2-Table 5_Vacuum Loading/0080046/0080046条纹图/008004640000.bmp]

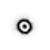

Supplement: S1 Dataset — (ZIP) [file pone.0327318.s001.zip › Minimal data set/Data for Table 2-Table 5_Vacuum Loading/0080046/0080046条纹图/008004640000.png]

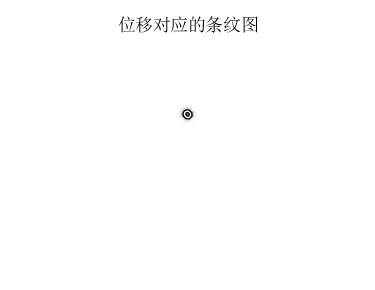

Supplement: S1 Dataset — (ZIP) [file pone.0327318.s001.zip › Minimal data set/Data for Table 2-Table 5_Vacuum Loading/0080046/0080046条纹图/008004650000.bmp]

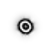

Supplement: S1 Dataset — (ZIP) [file pone.0327318.s001.zip › Minimal data set/Data for Table 2-Table 5_Vacuum Loading/0080046/0080046条纹图/008004650000.png]

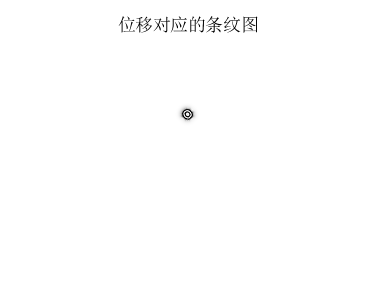

Supplement: S1 Dataset — (ZIP) [file pone.0327318.s001.zip › Minimal data set/Data for Table 2-Table 5_Vacuum Loading/0080046/0080046条纹图/008004660000.bmp]

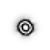

Supplement: S1 Dataset — (ZIP) [file pone.0327318.s001.zip › Minimal data set/Data for Table 2-Table 5_Vacuum Loading/0080046/0080046条纹图/008004660000.png]

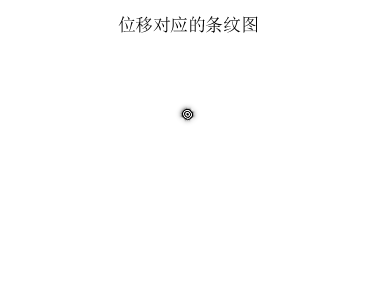

Supplement: S1 Dataset — (ZIP) [file pone.0327318.s001.zip › Minimal data set/Data for Table 2-Table 5_Vacuum Loading/0080046/0080046条纹图/008004670000.bmp]

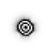

Supplement: S1 Dataset — (ZIP) [file pone.0327318.s001.zip › Minimal data set/Data for Table 2-Table 5_Vacuum Loading/0080046/0080046条纹图/008004670000.png]

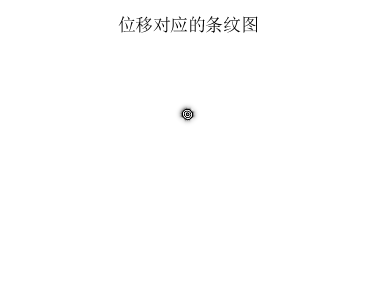

Supplement: S1 Dataset — (ZIP) [file pone.0327318.s001.zip › Minimal data set/Data for Table 2-Table 5_Vacuum Loading/0080046/0080046条纹图/008004680000.bmp]

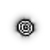

Supplement: S1 Dataset — (ZIP) [file pone.0327318.s001.zip › Minimal data set/Data for Table 2-Table 5_Vacuum Loading/0080046/0080046条纹图/008004680000.png]

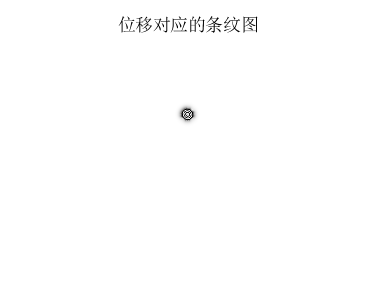

Supplement: S1 Dataset — (ZIP) [file pone.0327318.s001.zip › Minimal data set/Data for Table 2-Table 5_Vacuum Loading/0080046/0080046条纹图/008004690000.bmp]

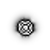

Supplement: S1 Dataset — (ZIP) [file pone.0327318.s001.zip › Minimal data set/Data for Table 2-Table 5_Vacuum Loading/0080046/0080046条纹图/008004690000.png]

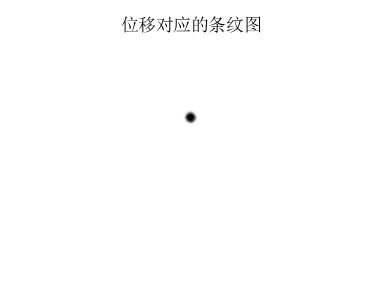

Supplement: S1 Dataset — (ZIP) [file pone.0327318.s001.zip › Minimal data set/Data for Table 2-Table 5_Vacuum Loading/010045/条纹图/01004510000.bmp]

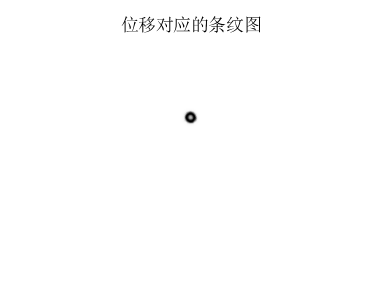

Supplement: S1 Dataset — (ZIP) [file pone.0327318.s001.zip › Minimal data set/Data for Table 2-Table 5_Vacuum Loading/010045/条纹图/01004515000.bmp]

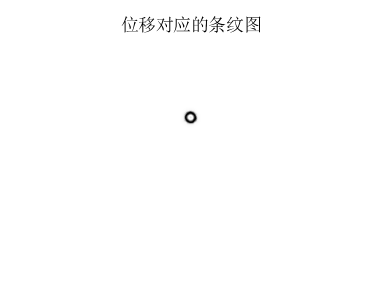

Supplement: S1 Dataset — (ZIP) [file pone.0327318.s001.zip › Minimal data set/Data for Table 2-Table 5_Vacuum Loading/010045/条纹图/01004520000.bmp]

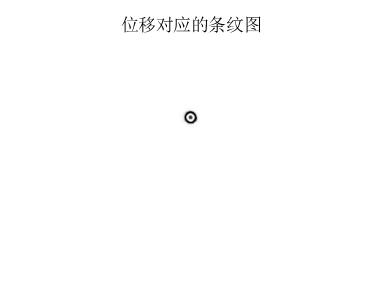

Supplement: S1 Dataset — (ZIP) [file pone.0327318.s001.zip › Minimal data set/Data for Table 2-Table 5_Vacuum Loading/010045/条纹图/01004525000.bmp]

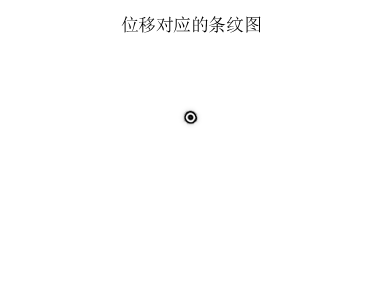

Supplement: S1 Dataset — (ZIP) [file pone.0327318.s001.zip › Minimal data set/Data for Table 2-Table 5_Vacuum Loading/010045/条纹图/01004530000.bmp]

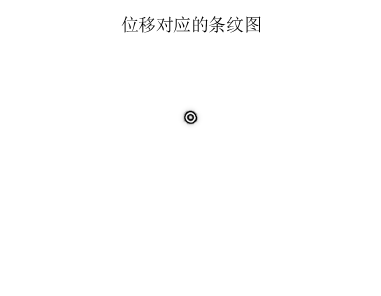

Supplement: S1 Dataset — (ZIP) [file pone.0327318.s001.zip › Minimal data set/Data for Table 2-Table 5_Vacuum Loading/010045/条纹图/01004535000.bmp]

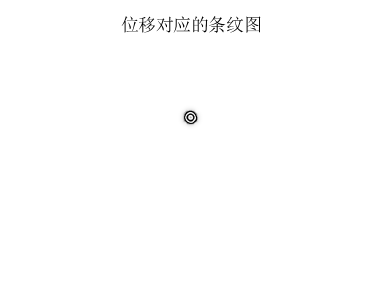

Supplement: S1 Dataset — (ZIP) [file pone.0327318.s001.zip › Minimal data set/Data for Table 2-Table 5_Vacuum Loading/010045/条纹图/01004540000.bmp]

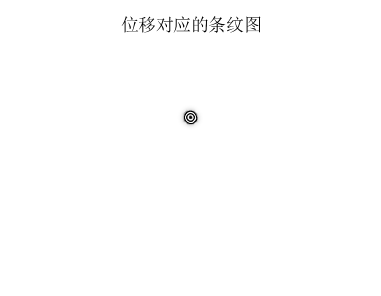

Supplement: S1 Dataset — (ZIP) [file pone.0327318.s001.zip › Minimal data set/Data for Table 2-Table 5_Vacuum Loading/010045/条纹图/01004545000.bmp]

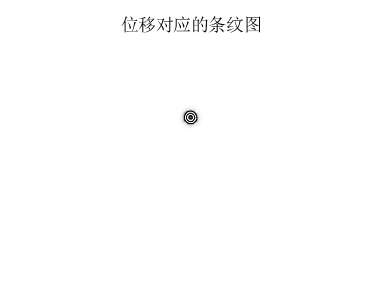

Supplement: S1 Dataset — (ZIP) [file pone.0327318.s001.zip › Minimal data set/Data for Table 2-Table 5_Vacuum Loading/010045/条纹图/01004550000.bmp]

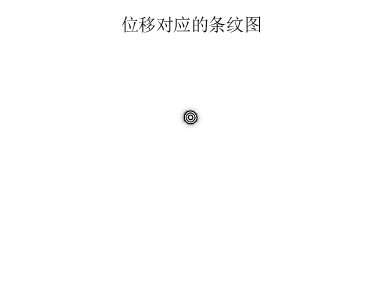

Supplement: S1 Dataset — (ZIP) [file pone.0327318.s001.zip › Minimal data set/Data for Table 2-Table 5_Vacuum Loading/010045/条纹图/01004555000.bmp]

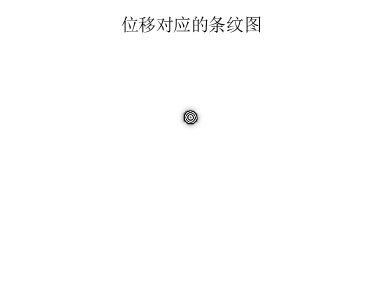

Supplement: S1 Dataset — (ZIP) [file pone.0327318.s001.zip › Minimal data set/Data for Table 2-Table 5_Vacuum Loading/010045/条纹图/01004560000.bmp]

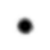

Supplement: S1 Dataset — (ZIP) [file pone.0327318.s001.zip › Minimal data set/Data for Table 2-Table 5_Vacuum Loading/010045/条纹图/10000.png]

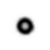

Supplement: S1 Dataset — (ZIP) [file pone.0327318.s001.zip › Minimal data set/Data for Table 2-Table 5_Vacuum Loading/010045/条纹图/15000.png]

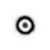

Supplement: S1 Dataset — (ZIP) [file pone.0327318.s001.zip › Minimal data set/Data for Table 2-Table 5_Vacuum Loading/010045/条纹图/25000.png]

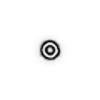

Supplement: S1 Dataset — (ZIP) [file pone.0327318.s001.zip › Minimal data set/Data for Table 2-Table 5_Vacuum Loading/010045/条纹图/35000.png]

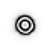

Supplement: S1 Dataset — (ZIP) [file pone.0327318.s001.zip › Minimal data set/Data for Table 2-Table 5_Vacuum Loading/010045/条纹图/40000.png]

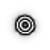

Supplement: S1 Dataset — (ZIP) [file pone.0327318.s001.zip › Minimal data set/Data for Table 2-Table 5_Vacuum Loading/010045/条纹图/50000.png]

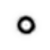

Supplement: S1 Dataset — (ZIP) [file pone.0327318.s001.zip › Minimal data set/Data for Table 2-Table 5_Vacuum Loading/010045/条纹图/Image 001.png]

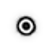

Supplement: S1 Dataset — (ZIP) [file pone.0327318.s001.zip › Minimal data set/Data for Table 2-Table 5_Vacuum Loading/010045/条纹图/Image 30000.png]

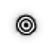

Supplement: S1 Dataset — (ZIP) [file pone.0327318.s001.zip › Minimal data set/Data for Table 2-Table 5_Vacuum Loading/010045/条纹图/Image 45000.png]

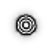

Supplement: S1 Dataset — (ZIP) [file pone.0327318.s001.zip › Minimal data set/Data for Table 2-Table 5_Vacuum Loading/010045/条纹图/Image 55000.png]

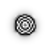

Supplement: S1 Dataset — (ZIP) [file pone.0327318.s001.zip › Minimal data set/Data for Table 2-Table 5_Vacuum Loading/010045/条纹图/Image 60000.png]

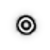

Supplement: S1 Dataset — (ZIP) [file pone.0327318.s001.zip › Minimal data set/Data for Table 2-Table 5_Vacuum Loading/010045/条纹图/iii35000.png]

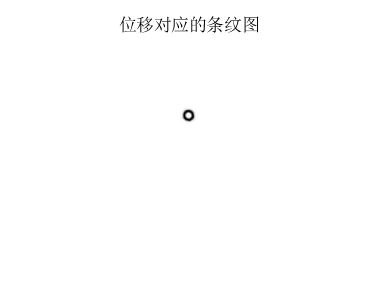

Supplement: S1 Dataset — (ZIP) [file pone.0327318.s001.zip › Minimal data set/Data for Table 2-Table 5_Vacuum Loading/010046/010046/01004610000.bmp]

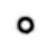

Supplement: S1 Dataset — (ZIP) [file pone.0327318.s001.zip › Minimal data set/Data for Table 2-Table 5_Vacuum Loading/010046/010046/01004610000.png]

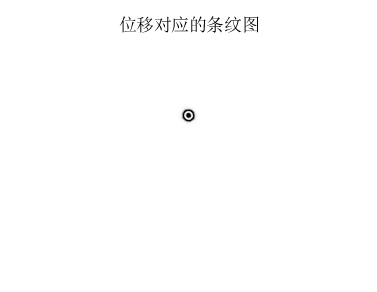

Supplement: S1 Dataset — (ZIP) [file pone.0327318.s001.zip › Minimal data set/Data for Table 2-Table 5_Vacuum Loading/010046/010046/01004615000.bmp]

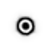

Supplement: S1 Dataset — (ZIP) [file pone.0327318.s001.zip › Minimal data set/Data for Table 2-Table 5_Vacuum Loading/010046/010046/01004615000.png]

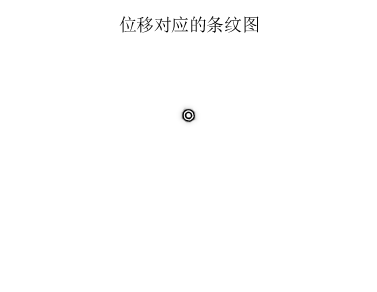

Supplement: S1 Dataset — (ZIP) [file pone.0327318.s001.zip › Minimal data set/Data for Table 2-Table 5_Vacuum Loading/010046/010046/01004620000.bmp]

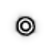

Supplement: S1 Dataset — (ZIP) [file pone.0327318.s001.zip › Minimal data set/Data for Table 2-Table 5_Vacuum Loading/010046/010046/01004620000.png]

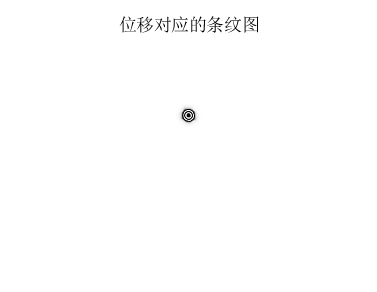

Supplement: S1 Dataset — (ZIP) [file pone.0327318.s001.zip › Minimal data set/Data for Table 2-Table 5_Vacuum Loading/010046/010046/01004625000.bmp]

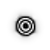

Supplement: S1 Dataset — (ZIP) [file pone.0327318.s001.zip › Minimal data set/Data for Table 2-Table 5_Vacuum Loading/010046/010046/01004625000.png]

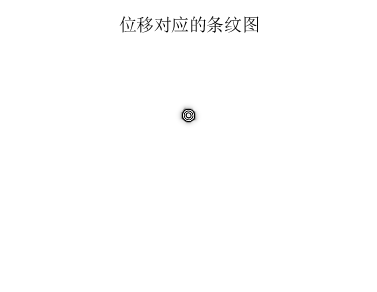

Supplement: S1 Dataset — (ZIP) [file pone.0327318.s001.zip › Minimal data set/Data for Table 2-Table 5_Vacuum Loading/010046/010046/01004630000.bmp]

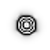

Supplement: S1 Dataset — (ZIP) [file pone.0327318.s001.zip › Minimal data set/Data for Table 2-Table 5_Vacuum Loading/010046/010046/01004630000.png]

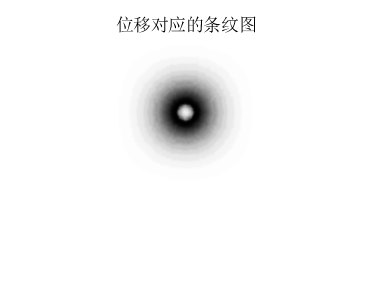

Supplement: S1 Dataset — (ZIP) [file pone.0327318.s001.zip › Minimal data set/Data for Table 2-Table 5_Vacuum Loading/010047/热加载条纹图/01004535.bmp]

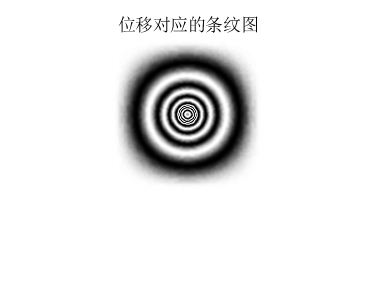

Supplement: S1 Dataset — (ZIP) [file pone.0327318.s001.zip › Minimal data set/Data for Table 2-Table 5_Vacuum Loading/010047/热加载条纹图/01004880.bmp]

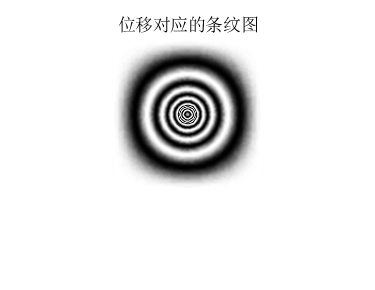

Supplement: S1 Dataset — (ZIP) [file pone.0327318.s001.zip › Minimal data set/Data for Table 2-Table 5_Vacuum Loading/010047/热加载条纹图/01004885.bmp]

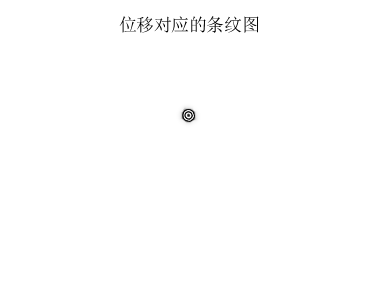

Supplement: S1 Dataset — (ZIP) [file pone.0327318.s001.zip › Minimal data set/Data for Table 2-Table 5_Vacuum Loading/010047/真空条纹图/01004710000.bmp]

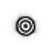

Supplement: S1 Dataset — (ZIP) [file pone.0327318.s001.zip › Minimal data set/Data for Table 2-Table 5_Vacuum Loading/010047/真空条纹图/01004710000.png]

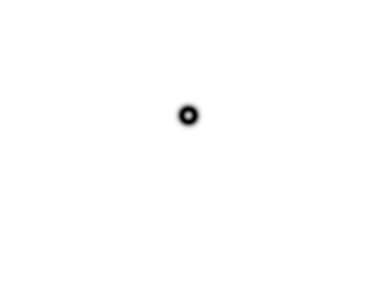

Supplement: S1 Dataset — (ZIP) [file pone.0327318.s001.zip › Minimal data set/Data for Table 2-Table 5_Vacuum Loading/010047/真空条纹图/01004713000.bmp]

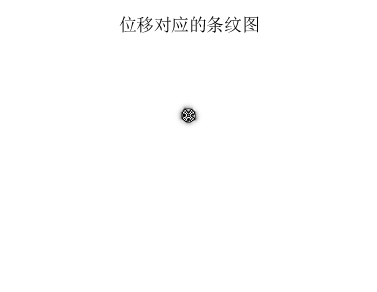

Supplement: S1 Dataset — (ZIP) [file pone.0327318.s001.zip › Minimal data set/Data for Table 2-Table 5_Vacuum Loading/010047/真空条纹图/01004715000.bmp]

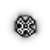

Supplement: S1 Dataset — (ZIP) [file pone.0327318.s001.zip › Minimal data set/Data for Table 2-Table 5_Vacuum Loading/010047/真空条纹图/01004715000.png]

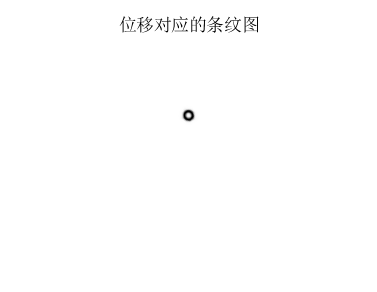

Supplement: S1 Dataset — (ZIP) [file pone.0327318.s001.zip › Minimal data set/Data for Table 2-Table 5_Vacuum Loading/010047/真空条纹图/0100474000.bmp]

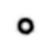

Supplement: S1 Dataset — (ZIP) [file pone.0327318.s001.zip › Minimal data set/Data for Table 2-Table 5_Vacuum Loading/010047/真空条纹图/0100474000.png]

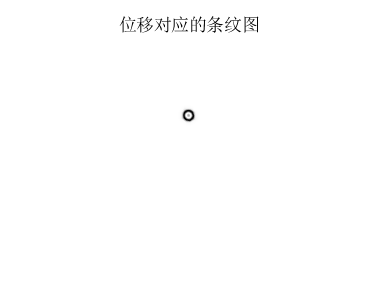

Supplement: S1 Dataset — (ZIP) [file pone.0327318.s001.zip › Minimal data set/Data for Table 2-Table 5_Vacuum Loading/010047/真空条纹图/0100475000.bmp]

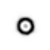

Supplement: S1 Dataset — (ZIP) [file pone.0327318.s001.zip › Minimal data set/Data for Table 2-Table 5_Vacuum Loading/010047/真空条纹图/0100475000.png]

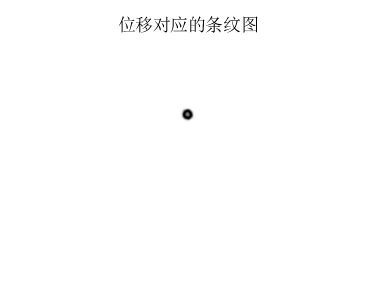

Supplement: S1 Dataset — (ZIP) [file pone.0327318.s001.zip › Minimal data set/Data for Table 2-Table 5_Vacuum Loading/010048/010048/0100481000.bmp]

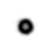

Supplement: S1 Dataset — (ZIP) [file pone.0327318.s001.zip › Minimal data set/Data for Table 2-Table 5_Vacuum Loading/010048/010048/0100481000.png]

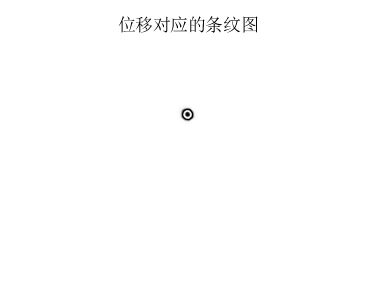

Supplement: S1 Dataset — (ZIP) [file pone.0327318.s001.zip › Minimal data set/Data for Table 2-Table 5_Vacuum Loading/010048/010048/0100482000.bmp]

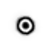

Supplement: S1 Dataset — (ZIP) [file pone.0327318.s001.zip › Minimal data set/Data for Table 2-Table 5_Vacuum Loading/010048/010048/0100482000.png]

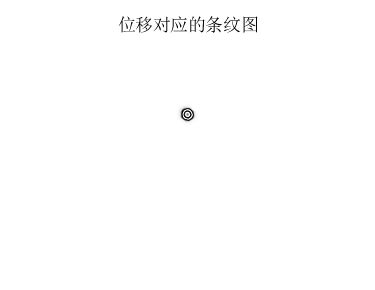

Supplement: S1 Dataset — (ZIP) [file pone.0327318.s001.zip › Minimal data set/Data for Table 2-Table 5_Vacuum Loading/010048/010048/0100483000.bmp]

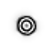

Supplement: S1 Dataset — (ZIP) [file pone.0327318.s001.zip › Minimal data set/Data for Table 2-Table 5_Vacuum Loading/010048/010048/0100483000.png]

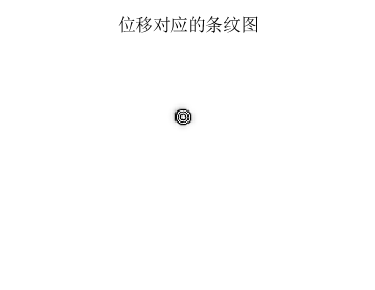

Supplement: S1 Dataset — (ZIP) [file pone.0327318.s001.zip › Minimal data set/Data for Table 2-Table 5_Vacuum Loading/010048/010048/0100484000.bmp]

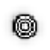

Supplement: S1 Dataset — (ZIP) [file pone.0327318.s001.zip › Minimal data set/Data for Table 2-Table 5_Vacuum Loading/010048/010048/0100484000.png]

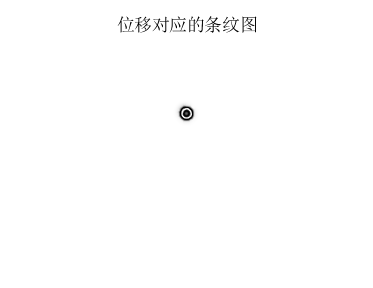

Supplement: S1 Dataset — (ZIP) [file pone.0327318.s001.zip › Minimal data set/Data for Table 2-Table 5_Vacuum Loading/0120046/0120046条纹图/012004610000.bmp]

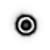

Supplement: S1 Dataset — (ZIP) [file pone.0327318.s001.zip › Minimal data set/Data for Table 2-Table 5_Vacuum Loading/0120046/0120046条纹图/012004610000.png]

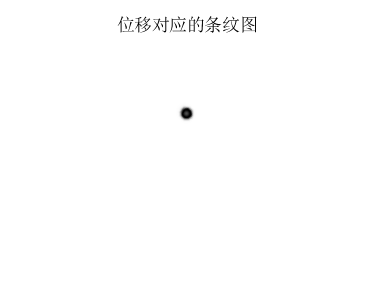

Supplement: S1 Dataset — (ZIP) [file pone.0327318.s001.zip › Minimal data set/Data for Table 2-Table 5_Vacuum Loading/0120046/0120046条纹图/01200464000.bmp]

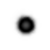

Supplement: S1 Dataset — (ZIP) [file pone.0327318.s001.zip › Minimal data set/Data for Table 2-Table 5_Vacuum Loading/0120046/0120046条纹图/01200464000.png]

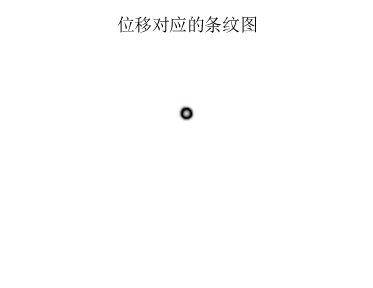

Supplement: S1 Dataset — (ZIP) [file pone.0327318.s001.zip › Minimal data set/Data for Table 2-Table 5_Vacuum Loading/0120046/0120046条纹图/01200465000.bmp]

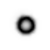

Supplement: S1 Dataset — (ZIP) [file pone.0327318.s001.zip › Minimal data set/Data for Table 2-Table 5_Vacuum Loading/0120046/0120046条纹图/01200465000.png]

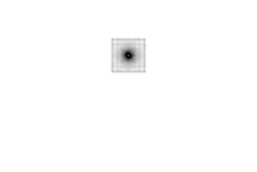

Supplement: S1 Dataset — (ZIP) [file pone.0327318.s001.zip › Minimal data set/Data for Table 6-Table 10_Thermal loading/1250-3-50/1250D10S05.bmp]

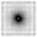

Supplement: S1 Dataset — (ZIP) [file pone.0327318.s001.zip › Minimal data set/Data for Table 6-Table 10_Thermal loading/1250-3-50/1250D10S05.png]

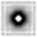

Supplement: S1 Dataset — (ZIP) [file pone.0327318.s001.zip › Minimal data set/Data for Table 6-Table 10_Thermal loading/1250-3-50/1250D15S05.png]

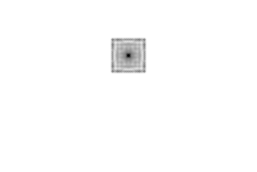

Supplement: S1 Dataset — (ZIP) [file pone.0327318.s001.zip › Minimal data set/Data for Table 6-Table 10_Thermal loading/1250-3-50/1250D5S05.bmp]

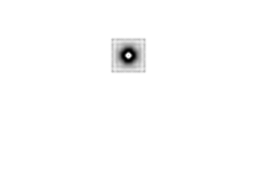

Supplement: S1 Dataset — (ZIP) [file pone.0327318.s001.zip › Minimal data set/Data for Table 6-Table 10_Thermal loading/1250-3-50/D15S05.bmp]

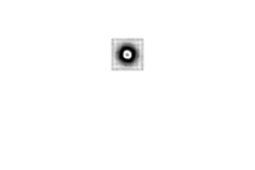

Supplement: S1 Dataset — (ZIP) [file pone.0327318.s001.zip › Minimal data set/Data for Table 6-Table 10_Thermal loading/1250-3-50/D20S05.bmp]

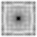

Supplement: S1 Dataset — (ZIP) [file pone.0327318.s001.zip › Minimal data set/Data for Table 6-Table 10_Thermal loading/1250-3-50/D5S05.png]

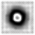

Supplement: S1 Dataset — (ZIP) [file pone.0327318.s001.zip › Minimal data set/Data for Table 6-Table 10_Thermal loading/1250-3-50/d20s05.png]

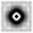

Supplement: S1 Dataset — (ZIP) [file pone.0327318.s001.zip › Minimal data set/Data for Table 6-Table 10_Thermal loading/1250-3-50/d20s05da.png]

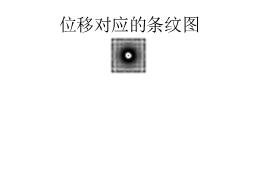

Supplement: S1 Dataset — (ZIP) [file pone.0327318.s001.zip › Minimal data set/Data for Table 6-Table 10_Thermal loading/2500-3S-50S/D10S05.bmp]

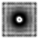

Supplement: S1 Dataset — (ZIP) [file pone.0327318.s001.zip › Minimal data set/Data for Table 6-Table 10_Thermal loading/2500-3S-50S/D10S05.png]

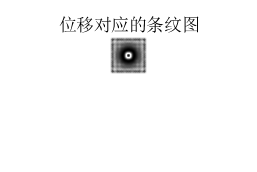

Supplement: S1 Dataset — (ZIP) [file pone.0327318.s001.zip › Minimal data set/Data for Table 6-Table 10_Thermal loading/2500-3S-50S/D11S05.bmp]

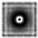

Supplement: S1 Dataset — (ZIP) [file pone.0327318.s001.zip › Minimal data set/Data for Table 6-Table 10_Thermal loading/2500-3S-50S/D11S05.png]

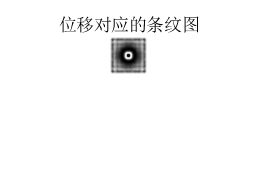

Supplement: S1 Dataset — (ZIP) [file pone.0327318.s001.zip › Minimal data set/Data for Table 6-Table 10_Thermal loading/2500-3S-50S/D12S05.bmp]

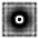

Supplement: S1 Dataset — (ZIP) [file pone.0327318.s001.zip › Minimal data set/Data for Table 6-Table 10_Thermal loading/2500-3S-50S/D12S05.png]

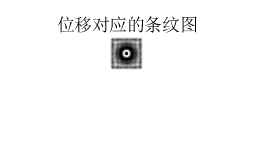

Supplement: S1 Dataset — (ZIP) [file pone.0327318.s001.zip › Minimal data set/Data for Table 6-Table 10_Thermal loading/2500-3S-50S/D13-S05.bmp]

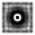

Supplement: S1 Dataset — (ZIP) [file pone.0327318.s001.zip › Minimal data set/Data for Table 6-Table 10_Thermal loading/2500-3S-50S/D13S05.png]

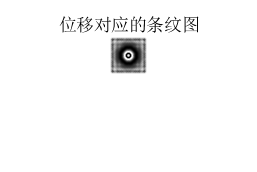

Supplement: S1 Dataset — (ZIP) [file pone.0327318.s001.zip › Minimal data set/Data for Table 6-Table 10_Thermal loading/2500-3S-50S/D14-S05.bmp]

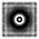

Supplement: S1 Dataset — (ZIP) [file pone.0327318.s001.zip › Minimal data set/Data for Table 6-Table 10_Thermal loading/2500-3S-50S/D14S05.png]
